# Left Chronic Otitis Media with Squamous Cell Carcinoma of the Middle Ear and Postauricular Mastoid Fistula: A Case Report

**DOI:** 10.31729/jnma.4954

**Published:** 2020-05

**Authors:** Shreedhar Prasad Acharya, Chetana Pathak, Sandarva Giri, Meera Bista, Deependra Mandal

**Affiliations:** 1 Kathmandu Medical College and Teaching Hospital, Kathmandu, Nepal; 2 Department of Ear, Nose and Throat, Kathmandu Medical College and Teaching Hospital, Kathmandu, Nepal

**Keywords:** *fistula*, *mastoid*, *middle ear*, *squamous cell carcinoma*

## Abstract

Though squamous cell carcinoma is the commonest tumor, it rarely presents in the middle ear and follows a history of chronic ear discharge. Postauricular mastoid fistula is also a rare complication of chronic otitis media. These two pathological changes occurring simultaneously are even rarer. We report a rare case of a 37 years old male with a history of left-sided chronic ear discharge and conductive hearing loss. Squamous cell carcinoma along with postauricular mastoid fistula was diagnosed based on high-resolution computed tomography scan and histopathology. Left modified radical mastoidectomy with tympanic membrane grafting was done under general anesthesia. Often middle ear tumor is associated with long-standing ear discharge and this case is a very good example. The co-occurrence of the middle ear tumor with mastoid fistula is extremely rare as both the entities are rare in itself.

## INTRODUCTION

Early presentation of middle ear squamous cell carcinoma (MESCC) is similar to that of chronic suppurative otitis media which may cover up MESCC.^1^ Postauricular mastoid fistula (PAMF) is an unusual complication of chronic otitis media or mastoid surgery and is known to complicate extensive meatoplasty and mastoidectomy.^2^ Patients with PAMF often seek medical assistance for closure due to cosmetic reasons.^2^

The postauricular mastoid fistula along with tumor in the middle ear is extremely rare. We hereby report the case of a 37-year old male patient with left middle ear tumor and left postauricular mastoid fistula that might have arisen as a complication of chronic otitis media.

## CASE REPORT

A 37-year male presented to ENT OPD with complaints of left ear discharge and decreased hearing since childhood. He gave the history of postauricular swelling 25 years back and started having discharge on the incision, which was insidious, continuous, scanty, mucopurulent with foul-smelling which was relieved with topical medications and had not used antibiotics for ear discharge till date. The discharge was aggravated since the last 4-5 months before presentation. He also gave a history of hearing loss which was insidious, progressive with difficulty in understanding phone conversation but can hear normal conversation sound. Past medical and surgical history was insignificant.

**Figure 1:**
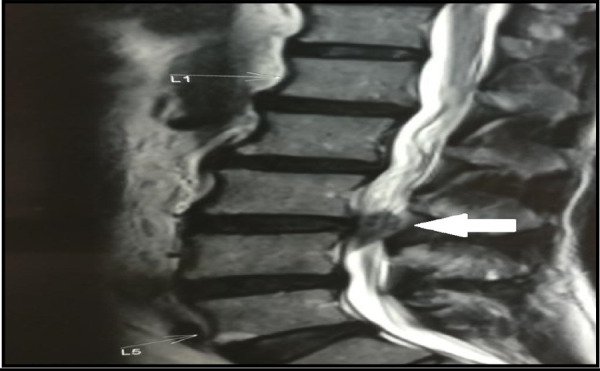
Postauricular fistulous opening of 1.5x 1.5 cm^2^.

On examination of the left ear, the fistulous opening of 1.51.5 cm^2^over the postauricular region erythematous surrounding filled with mucopurulent discharge and obliterated retro auricular groove was present. On cleaning the discharge, granulation was present which completely obliterated the opening of the fistula and external auditory canal (EAC) (Figure 1).

Normal platelet and hemoglobin level and total lymphocyte count of 10,100 mm^3^ with neutrophils predominant were seen. The serology was negative. The biochemistry report showed normal blood glucose, potassium, sodium, and creatinine level with slightly raised blood urea (23 mg/dl).

**Figure 2:**
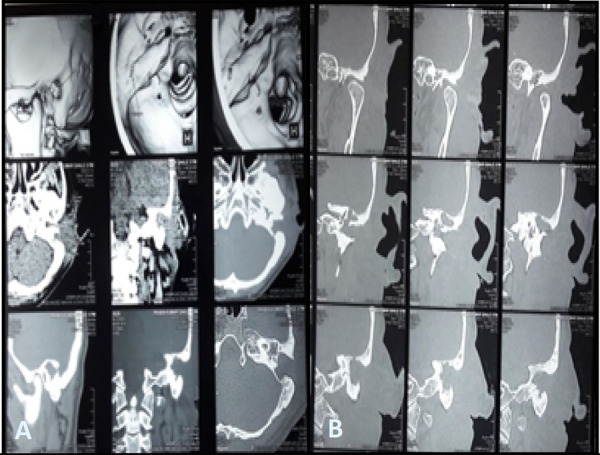
A,B. HRCT scan of temporal bone shows mastoid fistula and middle ear tumor.

Plain high resolution computed tomography (HRCT) scan of temporal bone showed soft tissue density and fluid in the left middle ear cavity involving epitympanum, mesotympanum, and hypotympanum. Soft tissue debris/fluid in left mastoid aditus and antrum with loss of pneumatization of left mastoid air cells helped us to make a diagnosis of a tumor in the middle ear. Erosion of the left mastoid outer cortex was also seen which suggests mastoid fistula. Focal erosion of left tegmen plate and left sigmoid sinus was present and only the part of left malleus was visualized while the rest of the ossicles were delineated (Figure 2 A, B). On audiological evaluation, moderately severe conductive hearing loss was observed in the left ear.

**Figure 3:**
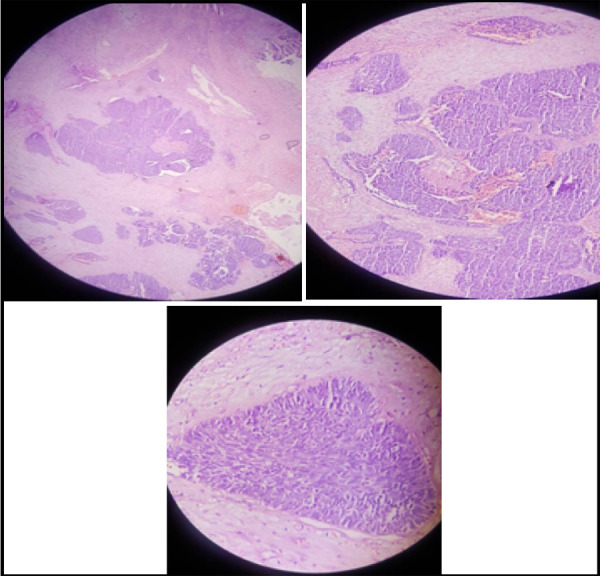
A,B,C. Histological slides of tissue on 4x, 10x, 40x magnification showing the features of squamous cell carcinoma.

The granulation tissue from the mastoid on the histopathological section shows tumor cells arranged in nests and islands infiltrating into the stroma, surrounded by desmoplastic reaction and mild to moderate nuclear pleomorphism. Prominent nucleoli, mitosis 2 to 6 per high power field, and some areas of hemorrhage and necrosis were also observed (Figure 3 A, B, C).

Modified radical mastoidectomy (MRM) with tympanic membrane graft was done under general anesthesia. An elliptical incision was made and the fistulous area was removed from the postauricular region. The findings seen in HRCT were also seen during the operative procedure.

Mastoidectomy cavity was present, polypoidal tissue was visualized over EAC, eroded ossicles, dehiscence over tegmen antri, and sigmoid sinus. The mastoid cavity was saucerized and graft was placed over a mastoidectomy cavity with a layer of gel foam under and as well as over the graft. over it. The umbilical tape was placed. Meatoplasty was done with suturing in multiple layers. At last, a bandage was applied over the mastoid and the facial nerve was assessed postoperatively which was found to be intact.

The patient was treated with IV fluids and IV antibiotics postoperatively and the daily dressing was done. Suture and umbilical tape were removed on the 7th postoperative day. The hospital stay period was uneventful. The patient was discharged with broad spectrum antibiotics administered with levocetrizine and muporicine ointment in mastoid area. He was followed after 7 days. The healing was accessed to be normal and the patient was referred to the cancer hospital for radiotherapy.

## DISCUSSION

Though squamous cell carcinoma is a common tumor but rarely occurs in the middle ear.^3^ The incidence of middle ear tumor is about 1/1,00,000.^4^ About 75 % of cases of squamous cell carcinoma has the history of long-term air discharge with a peak age at presentation being 60 years.^3^

The atypical symptoms and obscure signs frequently lead to misdiagnosis.^1^ Lasissi et al. reported a case of recurrent left ear discharge, which worsened six months before presentation.^5^ Though the history is quite close to our case the associated symptoms severe deep-seated left otalgia, left hemi-cranial headache, hearing loss, vertigo, and tinnitus, facial nerve palsy was absent in our case. Hu X-D reported a case with bloody discharge, painless skin ulceration in the mastoid area, where peripheral facial paralysis was seen,^1^ but in our patient, we only had an ulcerated mastoid region with mastoid fistula. MESCC generally has a poor prognosis as upon diagnosis most patients already have advanced disease. The interval from the onset of symptoms to definitive diagnosis can have a very wide range from 1 month to 60 years. Suzuki reported a range of least 3.5 years in three of the patients.^6^ Since our patient had an intact facial nerve and was diagnosed early so we have a chance of better prognosis. The correct diagnosis of middle ear pathologies requires a multidisciplinary approach.^7^ So, HRCT and histopathological correlation were done.

Postauricular mastoid fistula is one of the rare complications of chronic otitis media.^2^,^8^ Since the surrounding skin of mastoid fistula is mostly necrosed, it is difficult to get closed by primary closure.^9-10^ Mastoid fistula can also arise from the site incised for postauricular mass.

The most surprising part of our case was the presentation of the middle ear tumor along with a postauricular mastoid fistula. Also, our patient was below the mean age for the presentation of the middle ear tumor.

Surgery along with radiation therapy is the treatment approach for middle ear tumors. Colding-Wood et al. used radical mastoidectomy with pre- or postoperative irradiation for the treatment.^11^ But we performed a left MRM with a tympanic membrane graft to preserve the hearing with intact facial nerves and referred to the cancer hospital for post-operative radiation therapy. The surrounding skin of the mastoid area was removed at the beginning of the procedure.

The MESCC leads to serious complications, metastasize to the brain, or even result in the death of an individual. Though MESCC is a rare entity, it should be considered as a differential diagnosis of CSOM with hearing loss. HRCT and MRI are helpful for diagnosing middle ear diseases and should be considered for patients with long-term discharge and hearing loss. Early diagnosis and treatment should be done for a better prognosis. Closure of PAMF should also be done to prevent recurrent infection and not merely for cosmetic purposes.
